# Oh my aching gut: irritable bowel syndrome, *Blastocystis*, and asymptomatic infection

**DOI:** 10.1186/1756-3305-1-40

**Published:** 2008-10-21

**Authors:** Kenneth F Boorom, Huw Smith, Laila Nimri, Eric Viscogliosi, Gregory Spanakos, Unaiza Parkar, Lan-Hua Li, Xiao-Nong Zhou, Ülgen Z Ok, Saovanee Leelayoova, Morris S Jones

**Affiliations:** 1 *Blastocystis *Research Foundation, 5060 SW Philomath Blvd, #202, Corvallis, OR 97333, USA; 2 Scottish Parasite Diagnostic Laboratory, Stobhill Hospital, Glasgow, G21 3UW, UK; 3 Department of Medical Laboratory Sciences, Jordan University of Science & Technology, Irbid, Jordan 22110 (currently at Center for Disease Control, Atlanta, GA, USA); 4 Unite' Inserm U547, Institut Pasteur, 1 Rue du Professeury Calmette, BP 245, 59019 Lille Cedex, France; 5 Department of Parasitology, Entomology and Tropical Diseases, National School of Public Health, 196 Alexandras Ave, 11521 Athens, Greece; 6 WHO Collaborating Centre for the Molecular Epidemiology of Parasitic Infections and the State Agricultural Biotechnology Centre, School of Veterinary and Biomedical Sciences, Murdoch University, South Street, Western Australia 6150, Australia; 7 Department of Preventive Medicine, Weifang Medical University, 288 Shengli East Street, Shandong, Weifang, 261042, People's Republic of China; 8 National Institute of Parasitic Diseases, Chinese Center for Disease Control and Prevention, Shanghai, 200025, People's Republic of China; 9 Department of Parasitology, Faculty of Medicine, Celal Bayar University, Manisa, Turkey; 10 Department of Parasitology Phramongkutklao College of Medicine, Ratchathewi, Bangkok 10400, Thailand; 11 Clinical Investigation Facility, David Grant USAF Medical Center, 101 Bodin Circle, Travis AFB, CA 94535, USA

## Abstract

*Blastocystis *is a prevalent enteric protozoan that infects a variety of vertebrates. Infection with *Blastocystis *in humans has been associated with abdominal pain, diarrhea, constipation, fatigue, skin rash, and other symptoms. Researchers using different methods and examining different patient groups have reported asymptomatic infection, acute symptomatic infection, and chronic symptomatic infection. The variation in accounts has lead to disagreements concerning the role of *Blastocystis *in human disease, and the importance of treating it. A better understanding of the number of species of *Blastocystis *that can infect humans, along with realization of the limitations of the existing clinical laboratory diagnostic techniques may account for much of the disagreement. The possibility that disagreement was caused by the emergence of particular pathogenic variants of *Blastocystis *is discussed, along with the potential role of *Blastocystis *infection in irritable bowel syndrome (IBS). Findings are discussed concerning the role of protease-activated receptor-2 in enteric disease which may account for the presence of abdominal pain and diffuse symptoms in *Blastocystis *infection, even in the absence of fever and endoscopic findings. The availability of better diagnostic techniques and treatments for *Blastocystis *infection may be of value in understanding chronic gastrointestinal illness of unknown etiology.

## Review

### Introduction

*Blastocystis *is a prevalent enteric protist that infects a variety of vertebrates. Researchers have described asymptomatic and symptomatic infection in humans. Infection with *Blastocystis *is termed blastocystosis and has been associated with abdominal pain, diarrhea, constipation, fatigue[1], skin rash [2-4], and other symptoms. Although *Blastocystis *is commonly referred to as a protozoal parasite, small subunit (SSU) rRNA analysis has placed *Blastocystis *in the phylum Stramenopile, along with other organisms such as diatoms, brown algae, slime nets, and water moulds [2]. *Blastocystis *is non-motile, and is the only Stramenopile known to commonly cause infection in humans [2].

One of the earliest reports of symptomatic blastocystosis occurred in 1899 and was followed by sporadic reports through the 20^th ^century, that accelerated in 1984 [3]. In the United States, a 1987 survey by the Center for Disease Control showed the frequency of occurrence of *Blastocystis *in US clinical lab samples to be a relatively low 2.6% [4]. A study published by two physicians in California in 1988 reported symptoms of blastocystosis could usually be attributed to another cause and suggested that *Blastocystis *was non-pathogenic [5]. However, this finding was not in agreement with the experience of US researchers treating patients with international travel history [6,7] or with many researchers outside the United States [1,8].

A debate followed in 1990 between the Californian physicians and others in which some noted a lack of historical study identifying *Blastocystis *as pathogenic, and suggested that resolution of symptoms in patients following antiprotozoal therapy was due to the treatment of an undetected pathogen [9]. Objections notwithstanding, the clinical community has diagnosed and treated the infection frequently since then [6,10-12], although the research community describes *Blastocystis *pathogenicity as controversial [13]. *Blastocystis *is now by far the most prevalent mono-infection in symptomatic patients in the United States [14] and was found 28.5 times more often than *Giardia lamblia *as a mono-infection in symptomatic patients in a 2000 study [14]. Informal communications suggest the controversy surrounding this protozoan has made it an unpopular research topic in some countries (unpublished data, Kenneth Boorom).

### The problem of irritable bowel syndrome

**Figure 1:**
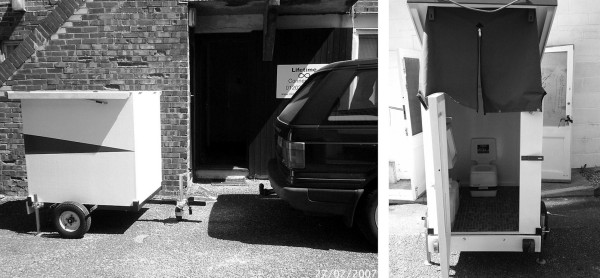
**Patients adapt to chronic gastrointestinal illness, which is found at an increasingly high rate in the United Kingdom**[**117**]. The "Mobilet" was developed by an IBS patient and consists of a toilet in an enclosed structure which can be towed behind a vehicle to facilitate travel by persons with severe chronic diarrhea. The UK journal *Gut Reaction *reported that the device sells for £1349 [19].

Irritable bowel syndrome (IBS) is a highly prevalent gastrointestinal disorder characterized by abdominal pain with diarrhea and/or constipation. The etiology of IBS has not been definitively established. IBS was originally thought to be a psychosomatic disorder [15], but more recent studies have identified chronic immune activation in IBS patients [16]. The annual direct and indirect costs of IBS in the United States may be as high as $30 billion [17], making it one of the most expensive gastrointestinal diseases in the US and other developed countries. Unlike viral and bacterial gastroenteritis, symptoms of IBS may last indefinitely [18]. The chronic nature of the disease may limit ordinary activities (Figure 1[19]). The disease raises health costs by 49% and much of the additional cost is due to extra-intestinal co-morbidities [20]. The prevalence of IBS in some developing countries is in the 35–43% range [21,22].

**Figure 2:**
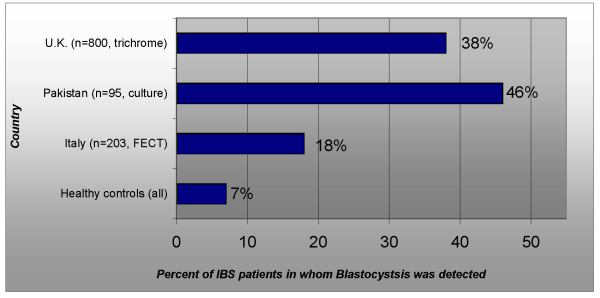
**Although IBS is ostensibly a functional disorder, IBS patients have been found to be infected with *****Blastocystis *****at statistically significant levels in Italy**[**37**]**, Pakistan**[**25**]**, the United Kingdom**[**24**,**26**]**but not Thailand **[**27**]. A study from Pakistan identified an elevated serum antibody response to *Blastocystis *in patients from whom *Blastocystis *could not be cultured [36]. The figure for the UK includes IBS and chronic GI illness. Numbers shown represent the total number of participants in the study (symptomatic and asymptomatic).

**Figure 3:**
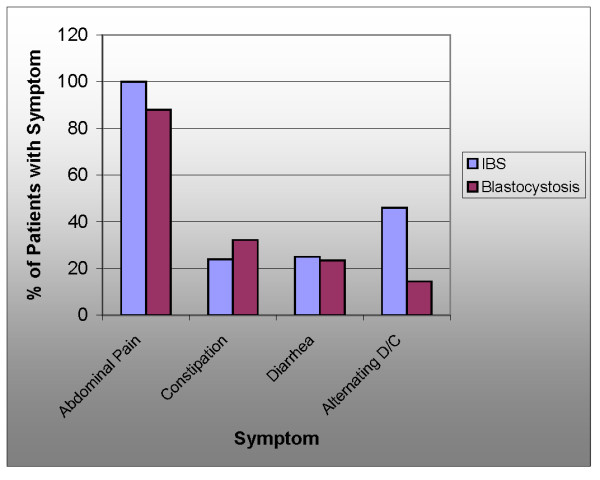
**Comparison of the frequency of symptoms seen in blastocystosis**[**1**]**to those seen in IBS**[**28**]. Host genetics may influence expression of symptoms in IBS [29].

**Figure 4:**
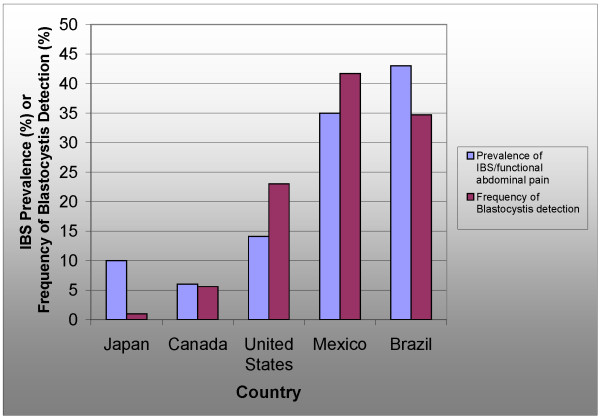
**Comparison of the prevalence of IBS and chronic abdominal pain to the frequency of detection of *****Blastocystis *****in Japan**[**22**,**30**]**, Canada**[**31**,**32**]**, United States**[**14**,**33**]**, Mexico**[**21**,**34**]**, and Brazil**[**22**,**35**].

In medical practice, a functional disorder can be described as "a set of symptoms not explained by structural or biochemical abnormalities" [23]. IBS is the only functional bowel disorder where a protozoal infection has been found in almost half of diagnosed cases using simple methods such as culture and staining (Figure 2[24-27]). The relative prevalence of symptoms of abdominal pain, diarrhea, and constipation in *Blastocystis *infection and IBS show a remarkable similarity (Figure 3[28,29]). High *Blastocystis *infection rates seem to accompany a high prevalence of IBS (Figure 4[30-35]). Researchers from Pakistan and other countries have suggested that IBS may be caused by *Blastocystis *[36,37]. However, the indeterminacy of *Blastocystis*' pathogenicity may have made it difficult to address this infection with a systematic approach used in other infectious diseases. The use of modern tools for identification and classification of *Blastocystis *isolates from humans, along with a better understanding of the pathogenesis of enteric protozoal infections may explain conflicting reports from researchers.

### Phylogeny

Following the discovery of *Blastocystis *in humans, it was assumed that humans carried a unique species of *Blastocystis*, which was given the name *Blastocystis hominis *[38]. Animals and birds were thought to carry different species, which were subsequently assigned names such as *Blastocystis ratti *for isolates from rats [39] and *Blastocystis galli *for isolates from chickens [40]. Phylogenetic analysis of SSU rRNA gene sequences of *Blastocystis *isolates from humans and animals has shown no evidence of a species of *Blastocystis *unique to humans [12]. Rather, humans acquire infection with the same species of *Blastocystis *acquired by rats, dogs, horses, cows, pigs, birds and other animals [12]. All nine of the subtypes of *Blastocystis *found in mammals and birds are found in humans as well. As a reflection of the low host specificity of *Blastocystis*, a classification system has been developed in which isolates are identified by subtype numbers rather than host names [41]. The nomenclature proposed describes such isolates as *Blastocystis *sp. subtype n where n is a number from 1 to 9. The genetic diversity that exists within the isolates previously identified as *Blastocystis hominis *is similar to the diversity that exists within the entire *Cryptosporidium *genus (Unpublished data, Dr. Eric Viscogliosi). The genomes of the mitochondrion-like organelle (MLO) of *Blastocystis *sp. subtypes 4 and 1 have been sequenced. The divergence seen between the MLO genomes of these two subtypes was greater than that seen between the MLO genomes of many congeneric species and even the MLO genomes from some species of differing genera [42].

**Figure 5:**
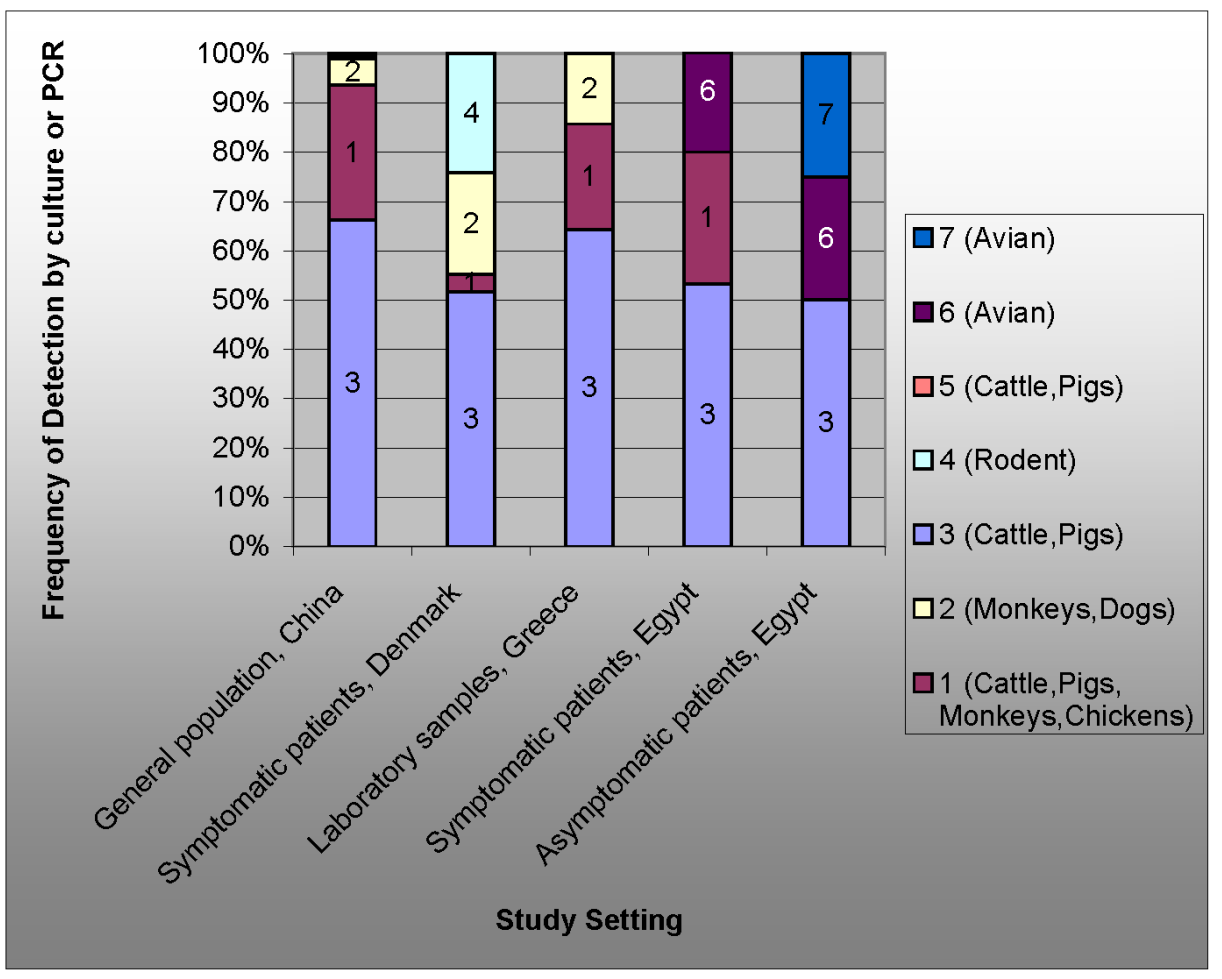
**Distribution of subtypes in*****Blastocystis*****found in the general Chinese population**[**43**]**, patients at a clinical lab in Denmark**[**44**]**, samples from a hospital in Greece**[**46**]**, patients in Egyptian lab **[**48**]. These subtypes are associated with various non-human hosts [12,74].

*Blastocystis *sp. subtypes 3 and 1 make up most chronic infections in humans [43-45] (Table 1 and Figure 5[46]). These types are carried by cows, pigs, chickens, and horses [12]. *Blastocystis *sp. subtypes 3 and 1 are also associated with most symptomatic mono-infections [44,47-49]. Researchers have identified *Blastocystis *sp. subtype 2 infection as frequently asymptomatic [50] or occurring primarily as a co-infection in symptomatic patients [44] or carried primarily by older adults [51]. Expression of symptoms does not equate with ease of detection – *Blastocystis *sp. subtype 2 is the most readily detected [44] while *Blastocystis *sp. subtype 3 is difficult to detect [52] and may remain undetected in symptomatic patients despite extensive laboratory testing [49].

**Table 1:** Based on a classification system published in January 2007, *Blastocystis *isolates from humans, animals, and birds are identified as *Blastocystis *sp. subtypes 1 to 9, as determined by analysis of SSU rRNA sequences [41].

**Sub-type #**	**Yoshikawa subtype # (used in some earlier studies) **[41]	**% of *Blastocystis *isolates in population belonging to this subtype from study in China adjusted for co-infections **[43]	**Results from animal study from Egypt **[48]	**Non-human carriers **[12,74]	**Characteristics in human infection**	**Detectability +=Difficult +++=Readily Detected **[44,74]
*Blastocystis *sp. subtype 3	(3)	66%	Illness	Cows, Pigs	Associated with most chronic symptomatic infections [44,47,51]	+

*Blastocystis *sp. subtype 1	(1)	28%	Illness Death	Cows, Pigs, Chickens, Monkeys	Associated with many chronic symptomatic infections [44,47,51]	++

*Blastocystis *sp. subtype 2	(5)	5%	Not done	Dogs, Monkeys	Usually asymptomatic [50] or co-infection in symptomatic patients [44]	+++

*Blastocystis *sp. subtype 4	(7)	0.5%	Not done	Rodents	Rare in one population study [43], but was more common in a study of symptomatic patients at clinic [52]	++

*Blastocystis *sp. subtype 6	(4)	0.5%	Illness	Birds	Rare in one population study [43], but found in a study of symptomatic patients at a clinic [48]	Unknown

*Blastocystis *sp. subtype 5	(6)	0%	Not done	Pigs	Rare in one population study [43]	Unknown

*Blastocystis *sp. subtype 7	(2)	0%	No Illness	Birds	Rare in one population study [43], but found in a study of asymptomatic individuals [48]	Unknown

China has completed the only large study on the prevalence of different *Blastocystis *subtypes in the general population, and that study found most infections were associated with subtypes 1, 2 and 3.

Subtypes 4, 6, and 7 appear to show some degree of host specificity, in that their only established non-human hosts are rodents (subtype 4) and birds (subtypes 6 and 7) [12]. This host specificity may be responsible for the low prevalence of these subtypes in studies of the human population [53], although they appear more frequently in clinical laboratory studies where samples are collected from symptomatic patients [48,52], suggesting the possibility that these may be associated with acute infection in humans. However, the number of studies performed to date is small, and additional research is needed to understand potential differences in the course of infection associated with various subtypes.

Some isolates from the American Type Culture Collection (ATCC) have been genotyped, and belong to subtype 1 (ATCC 50177, 50609, 50610, 50751, 50752), subtype 3 (ATCC 50587, 50629, 50754), subtype 4 (ATCC 50608, 50753) while ATCC 50588 and 50613 are mixed [54]. The ATCC's records show that the most recently accessioned strain of *Blastocystis *was acquired in 1995 [55].

### Symptoms

A 1989 Saudi study of over 200 symptomatic patients described symptoms of abdominal pain, constipation, diarrhea, nausea, and anorexia [1]. The study also noted a number of extra-intestinal symptoms, such as headaches, depression, and fatigue. The inclusion of these as symptoms was criticized in the 1980's [9], but subsequently found to be co-morbidities in IBS [56]. A 1991 study from a researcher at the US National Institutes of Health described the unusual clinical presentation of blastocystosis [3]:

"The most usual complaint of blastocystosis patients is of intense abdominal discomfort accompanied by pain. Diarrhea is not standard, and constipation is common. The symptoms gleaned from the literature include abdominal pain, discomfort, anorexia, bloating, cramps, diarrhea, constipation, alternating diarrhea and constipation, watery diarrhea, mucus diarrhea, vomiting, dehydration, sleeplessness, nausea, weight loss, inability to work, lassitude, dizziness, flatus, pruritis, and tenesmus. Blood in the stool as well as excessive mucus and leukocytes have been reported."

In a study from Jordan, the most common symptoms reported in preschool children were abdominal pain, recurrent diarrhea, cramps, anorexia, and fatigue [57]. In older school children, additional symptoms were seen, such as mild diarrhea, nausea, bloating, and alternating diarrhea and constipation [58]. A 1989 study of 16,545 specimens from a clinical laboratory in Vancouver, Canada found *Blastocystis *as a mono-infection in 342 patients [10]. In this study, the symptoms found in 143 patients whose physicians responded to a survey were diarrhea, pain, nausea, gas, malaise, fever, weight loss, and chills. A frequency of bowel movements ranging from 1 to 25 per day was noted. Of the 69 patients responding to a detailed survey, 8 reported bloody diarrhea [10]. In a smaller study of patients in Oregon with chronic gastrointestinal illness and findings of infection with *Blastocystis *sp. subtype 3, intermittent bloody diarrhea was reported by one patient as well [49].

Testimony to the Oregon State Legislature by physician-diagnosed patients with blastocystosis included descriptions of severe fatigue, with one patient noting the inability to walk more than 15 minutes [59]. Symptoms reported in a collection of reports from patients in the United States included those from a returnee from Nepal were described as "constipation, spasms in ascending colon area, large loss of weight, occasional loose stools, fatigue, mental fogginess, increase in symptoms after eating, the need to eat every few hours, fast heartbeat, pale skin and others [60]." Upper gastrointestinal symptoms, such as dyspepsia, are also seen in *Blastocystis *infection, particularly in females according to one study [51]. Food intolerance was noted in the original Saudi study [1]. Patients with blastocystosis may develop elaborate exclusion diets to control symptoms (unpublished data, Ken Boorom).

### Dermatological involvement

**Figure 6:**
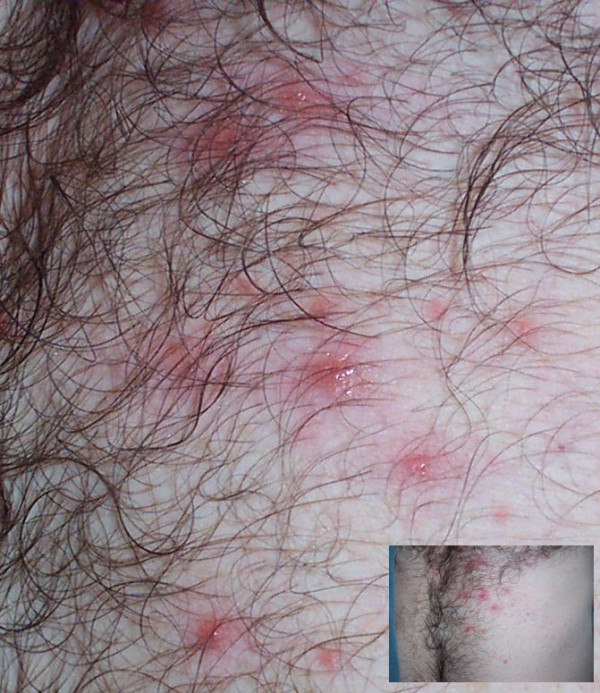
**Rash from 39-year old US male diagnosed with chronic blastocystosis acquired domestically**[**60**]. Skin rash in *Blastocystis *infection has been described as recurrent [65] and intensely pruritic [67]. Diagnosis of blastocystosis was by exclusion: eleven ova and parasite exams (trichrome staining) performed at clinical laboratories from 2003–2006 were negative except for findings of *Blastocystis*. Colonoscopy, endoscopy, and gluten challenge test were negative. The infection was unresponsive to metronidazole, nitazoxanide, and TMP/SMX. The isolate was genotyped as *Blastocystis *sp. subtype 3 in a 2007 study [49].

Reports of skin rash began appearing in the literature in 1993 [61], with additional reports in the following years [62-65]. Recent clinical studies have reported the association of skin rash with *Blastocystis *infection [51,66]. A Greek case report of one patient with *Blastocystis *associated rash identified the genotype infecting the patient as *Blastocystis *sp subtype 3 [67]. Dermatologists have found *Blastocystis *infection at a statistically significant rate in patients with allergic skin conditions [68]. Figure 6 show such a rash.

### Diagnostic techniques

Diagnosis of *Blastocystis *infection involves the detection of the organism, and a determination as to whether its presence is responsible for symptoms. Methods which have been used clinically and in research studies for this purpose are described below.

### Detection of the organism in stool specimens

Most clinical methods endeavor to identify infection by finding the organism in stool specimens. Variations on this method may involve concentration, staining, culturing, and extraction of DNA followed by polymerase chain reaction (PCR) testing. Most methods have been found to have low sensitivity [69] (Table 2).

**Table 2:** Sensitivities of detection of *Blastocystis *reported in various studies.

**Detection Method**	**compared to...**	**reported a sensitivity of**	**Year and country of study**
Formol ethyl acetate concentration technique (FECT)	Culture	0%	2004, UK [116]
FECT	Culture	19.6%	2002, Thailand [72]
Simple Smear	Culture	16.7%	2004, Thailand [69]
Simple Smear	Culture	42.5%	2002, Thailand [72]
Trichrome Staining	Culture	40.2%	2004, Thailand [69]
FECT	PCR	50%	2007, Denmark [44]
Sodium acetate-acetic acid-formalin (SAF) concentration technique	PCR	82%	2007, Denmark [44]
Culture	PCR	89%	2007, Denmark [44]
ELISA serum antibody	Culture	92.1%	2008, China [87]
Merthiolate-Iodine-Formalin	--	(no studies found)	

Sensitivities are representative only, and based on a small number of studies. Actual sensitivities may vary between laboratories due to variation in method, operator training, and quality of microscopic equipment [109], or variation in the distribution of *Blastocystis *subtypes being detected [44].

**Figure 7:**
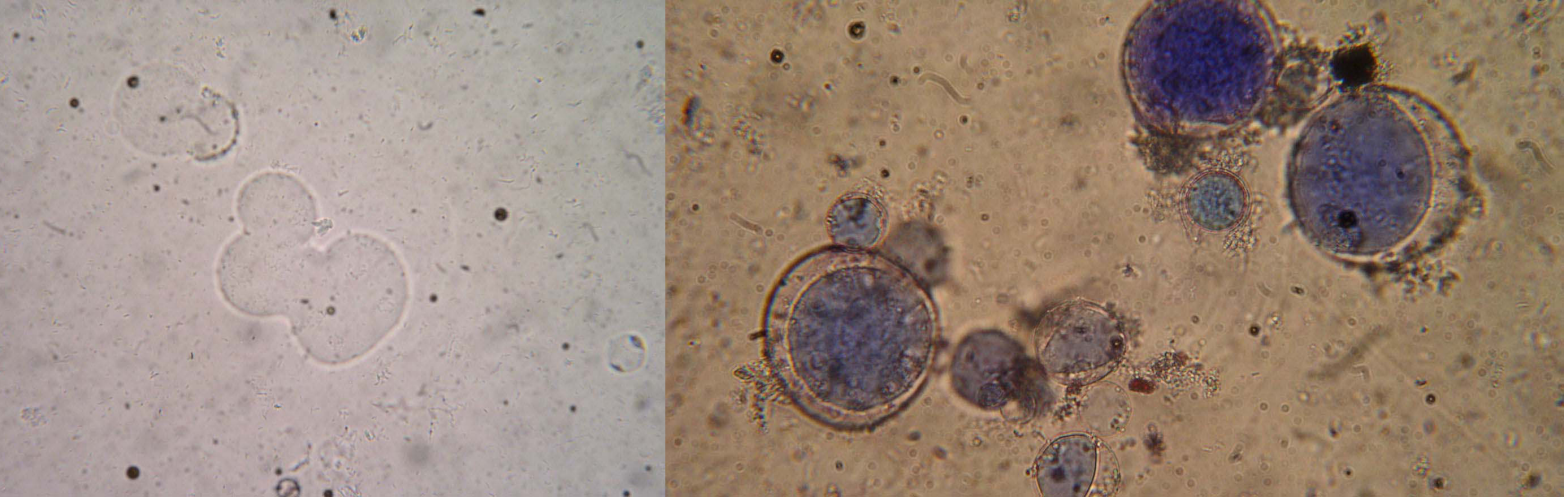
**(LEFT)*****Blastocystis*****in simple smear.** Researchers have cited the non-descript appearance of *Blastocystis *as one reason for the low sensitivity of clinical diagnostic methods, along with the possibility that not all stages have been documented [52]. (RIGHT) The classic diagnostic form of *Blastocystis *found in the stool of patients varies in size from 6 to 40 μm. The parasite is characterized by a central body (blue) that morphologically resembles a vacuole. The central body pushes the nuclei to the periphery of the cell. The central body is a reservoir for proteases [83] and may serve other functions as well. Photos courtesy of Dr. Hanaa Moussa, Cairo University.

*Blastocystis *is one of numerous human protozoan and metazoan intestinal parasites sought for in stools in clinical microbiology laboratories. Therefore, generic, physico-chemical methods such as the formol ethyl acetate concentration technique (FECT) will not necessarily maximize recoveries of all intestinal parasites because of their differing specific gravities and sensitivities to formalin. Both parasite morphology and morphometry may vary. Some isolates are only 6–8 μm in size [70] which makes microscopical detection difficult, especially when parasites are present in small numbers. Vegetative stages can be mistaken for fat globules, leukocytes, or other artifacts in the stool (Figure 7). The quantity of *Blastocystis *shed has been found to vary significantly between patients, and over time from the same experimental animal [71]. Infection with *Blastocystis *sp. subtype 3 is less readily detected than infection with *Blastocystis *sp. subtype 2 using FECT [44]. As *Blastocystis *sp. subtype 2 is less likely to be associated with symptomatic mono-infection [44,50], this effect may be responsible for producing conflicting results between researchers.

To improve detection rates, one study reported the use of a "purged sample" [6], which involves the use of a laxative. Stool culture has been suggested as an additional method, which may offer the best tradeoff between sensitivity and cost and may be less costly than methods currently in use [44]. Xenic cultures of *Blastocystis *can be produced from stool samples with inexpensive materials, and without the use of an anaerobic chamber [72]. The absolute sensitivity of stool culture as a diagnostic method is unknown. Stool culture may be positive in only 50–70% of infections with *Entamoeba histolytica *[73]. PCR testing of DNA extracted directly from stool specimens is considered to be the most sensitive method for the detection of *Blastocystis*, and represents a 10% improvement over culture [52]. It is currently the only way to differentiate subtypes of *Blastocystis*, and is used in research studies where genotyping is necessary [74-76]. A real time PCR test has been developed [54].

PCR testing of DNA extracted directly from stool specimens is a valuable tool for genotyping *Blastocystis *in specimens [74-76]. However, complexities associated with this method should be noted to guard against overconfidence in this as a diagnostic tool. Stool specimens contain PCR inhibitors which can interfere with the detection process. The high concentration of nucleases in some protozoa can interfere with detection [77]. *Blastocystis*' ability to undergo programmed cell death with associated DNA fragmentation [78] may also complicate PCR testing. Finding suitable loci in order to detect and differentiate isolates is a challenge because the entire genome of *Blastocystis *has not been sequenced and substantial genetic heterogeneity exists among the nine subtypes displayed within the genus. Stool samples have been observed to convert to PCR negative after remaining at room temperature for several hours, although the organism is still visible microscopically (unpublished data, Dr. Gregory Spanakos). Stool specimens have been found to convert to PCR negative after a few months of cold storage at -20C (personal communications, Dr. Morris S. Jones and Dr. Saovanee Leelayoova). Cryopreservation of extracted DNA may offer an alternative.

### Significance of detection of the organism in stool specimens

In addition to detection of *Blastocystis*, there is a need to determine if it is the cause of illness in patients. Some early reports suggested that *Blastocystis *might be diagnosed as a cause of illness only when present in large numbers in stool samples. *Blastocystis *is now the only protozoan whose presence is reported along with its quantity by US pathologists, with standardized terms such as "rare, few, moderate, and many" describing its abundance [79]. However, several studies have found no correlation between the quantity of *Blastocystis *in stool samples and symptoms [10,51,80]. Researchers have found that tests with low sensitivity failed to detect infection with the type of *Blastocystis *most frequently found in symptomatic patients [44]. Therefore, quantity of organisms identifiable in stool specimens may be a poor indicator of symptomatic status.

Genotyping of *Blastocystis *isolates may offer useful clinical information, especially as more studies are accumulated [44,47,51]. It may be possible to differentiate between infections which are likely to be asymptomatic, acutely symptomatic, and chronically symptomatic by subtyping *Blastocystis*. Isolates that have been associated with symptomatic infection in humans have also been found in asymptomatic carriers, so subtyping can not establish *Blastocystis *as a cause of illness in a particular patient.

One study has reported the ability to distinguish between symptomatic and asymptomatic infections by culturing stool samples and identifying specific forms in culture [81]. Because a limited number of isolates may have been used in that study, additional work would be needed to understand the applicability of this technique in other settings.

### Detecting other factors in stool specimens

Some diagnostic tests seek to identify factors in stool specimens which would be uniquely associated with symptomatic infection. These may offer better sensitivity than assays used to detect *Blastocystis *directly from stool.

One researcher reported success in the use of a fecal IgA assay to discriminate between symptomatic and asymptomatic *Blastocystis *infections [82]. In that study, the researcher reported that all patients with symptomatic *Blastocystis *infection tested positive at a titer of 1:400, while all asymptomatic *Blastocystis *carriers and uninfected patients tested negative at that titer.

As the pathogenesis of *Blastocystis *has been centered on proteases [83], one approach may be to detect the protease rather than the *Blastocystis*, on the assumption that *Blastocystis *may secrete higher levels of protease in symptomatic patients. Although it has not been applied with the intention of detecting blastocystosis, researchers have reported success in using a fecal serine protease assay in the study of diarrhea predominant irritable bowel syndrome (IBS-d) [84]. This study found that patients with IBS-d exhibited significantly higher protease levels in stool specimens, which were not found in patients diagnosed with diarrhea from acute infectious causes.

### Detecting serum antibody response

Serum antibody tests using whole cell antigen have been developed many times for research purposes, and generally show a correlation between serum antibody response and symptomatic blastocystosis [36,82,85-87]. A 2007 study from China found that the serum antibody test was almost as sensitive as stool culture [87]. Researchers have found this test to be highly selective and sensitive for symptomatic *Blastocystis *infection, although additional trials are needed [85]. One study reported the ability to differentiate between most symptomatic and asymptomatic carriers using an ELISA assay quantitatively [82]. A 1991 study noted an effort to make antisera available commercially [3]. A United States company has reported its investigation of this type of assay as a diagnostic product [88].

### Detecting infection through biopsies

In one study, colonic biopsies from IBS patients were found to produce elevated levels of serine protease [89]. Since the biopsies, but not necessarily the stool samples, seem to consistently contain the etiological element of such gastrointestinal illness, these may provide a better basis for the development of a "Gold Standard" for assays that detect infectious elements associated with IBS.

### How sensitive does an assay need to be?

Early studies suggested *Blastocystis *produced symptoms by "overgrowing" in the gastrointestinal tract, which lead to the practice of diagnosing blastocystosis only when the organism was found in large quantities in stool samples. A view more consistent with observed data suggests that specific genotypes of *Blastocystis *[48] interact with host factors, triggering an immune reaction [85] and producing proteases that produce symptoms [83]. As an extracellular enteric protozoan, *Blastocystis *is theoretically capable of completing its life cycle without damaging host tissue, so symptomatic infection may be considered to be a side effect of infection. The mechanism that produces the symptoms may differ from the mechanism by which *Blastocystis *reproduces and expresses itself in stool samples.

*Blastocystis *undergoes programmed cell death in response to antibody exposure [90-93], so it is possible that patient immune response may complicate recovery of *Blastocystis *from stool specimens. One study found that the inability to culture *Blastocystis *from patients was correlated with an IgG3 response [36]. That study also found that the ability to culture *Blastocystis *from symptomatic patients was poorly correlated with positive results in immunological testing [36]. The author attributed the effect to residual immune response from treated *Blastocystis*. An alternate possibility is that antibiotic treatment converts stool test results from positive to negative, but leaves patients with a residual infection.

### Treatment

Modern treatment of *Blastocystis *has generally been with metronidazole, with studies from the Middle East and US reporting treatment success [1,6]. Co-trimoxazole (TMP/SMX) has been used as well [94,95], as have nitazoxanide [96] and rifaximin [97].

Resistance to metronidazole was reported as early as 1991 [3]. Researchers have reported frequent resistance [98-100] and metronidazole may no longer be useful as a first line treatment [100]. Metronidazole resistance varies geographically [98] and Appendix A summarizes some resistance studies (see additional file 1). One clinic has reported success with a combination of secnidazole, nitazoxanide, and furazolidone [100]. All three drugs are not available in many countries. A 2006 text described a US patient returning from Nepal with chronic blastocystosis who was treated without success over a period of three years with iodoquinol, paromomycin, doxycycline, albendazole, tinidazole, ornidazole, quinacrine, nitazoxanide, rifaximin, furazolidone, co-trimoxazole, itraconazole, ketoconazole, and various combinations of those drugs [60]. A 1916 study described *Blastocystis *as an "infection that is difficult to get rid of" and noted the use of emetine [101].

A literature survey conducted for this review identified at least 79 studies describing treatment and epidemiology of *Blastocystis *(see additional file 1), but controlled investigation into the efficacy of drugs used in treatment is almost non-existent. Only two studies have screened drugs *in vitro *for effectiveness against *Blastocystis *[102,103]. The most recent *in vitro *study is over 17 years old, and no study has genotyped the *Blastocystis *isolates used, so it can not be determined if the isolates used in those studies are the same ones physicians seek to treat today.

The list of issues that complicates development of treatments for *Blastocystis *is long. Mouse models have existed since 1997 [104], but no animal studies have been published to evaluate treatment efficacy of drugs. The development of antiprotozoal resistance may be a factor in treatment failure, but there is a lack of evidence to show that this drug (or any other) should be effective against *Blastocystis*. Little is known about the organism's metabolic processes, although a recent study has identified the role of *Blastocystis' *mitochondrion-like-organelles in the reduction of ferrodoxins [105] that play a role in the conversion of metronidazole into its active state [106]. Metronidazole has been found to be one of the more effective drugs *in vitro *[102], but *in vitro *effectiveness may not correlate well with animal study in the treatment of parasitic infections [107]. Other factors in treatment failure, such as treatment compliance, remain uninvestigated. Some patients report an inability to complete metronidazole treatment due to vomiting, or difficulty administering the treatment to children (unpublished data, Kenneth Boorom).

No study has been published to identify agents that would be effective against the variants from IBS patient which exhibit metronidazole and furazolidone resistance *in vitro *[99]. Many drugs which have been reported to be effective against infection have been removed from common clinical use [3], although it is not known if those drugs would be effective in current infections. Many studies do not provide information about the length of time the patient had been symptomatic or provide follow-up information on the patient's condition. Most treatment studies lack information about the genotype being treated, so it is difficult to assess their significance. The lack of a reliable diagnostic method to determine if the infection has been eradicated complicates the evaluation of treatments.

### Prevalence

Before discussing the prevalence of *Blastocystis *in the population, several points of interest should be addressed. Because diagnostic methods vary widely in sensitivity, figures reported from studies may reflect the testing method as much as the actual frequency of occurrence. Statistics gathered from clinical diagnostic laboratories are often reported as "prevalence," but as laboratory specimens are often submitted with the intention of diagnosing an illness, this practice may overestimate the prevalence in the population. Such numbers should properly be reported as "frequency of detection of *Blastocystis *in laboratory samples by (method)."

### Prevalence in China

The only extensive studies of *Blastocystis *prevalence have been performed in China. A 2007 study evaluated 2321 samples taken from 4 geographic areas for *Blastocystis *presence and genotype (Figure 5) [43]. The prevalence of *Blastocystis *infection ranged from 1.9% in Shanghai municipality (in the East) to 32.6% in Menghai county (in the West).

### Frequency of detection in United States and Canada

It has been suggested that case reports of *Blastocystis *increased in frequency after 1984 [3]. An analysis of studies reporting the frequency of detection of *Blastocystis *from North American laboratories suggests an increasing trend that began after 1988, followed by a reducing trend that began around 2000 (see Appendix A in additional file 1). Controversy concerning *Blastocystis *may have been a reflection of the disease's emergence, as the debate peaked in the time period of 1988–1991, with a series of letters exchanged between researchers in the *Journal of Clinical Microbiology *[5,9,108-110].

### Changing genotypes

Studies from the 1980's and early 1990's from the Western United States and Canada indicated that *Blastocystis *was usually found as a co-infection, or was found in asymptomatic patients [5] and was comparatively rare [10,111]. These are characteristics associated with *Blastocystis *sp. subtype 2 [43,44,51]. A 2000 study found that *Blastocystis *was much more prevalent and it was no longer a co-infection in most patients which would be consistent with the characteristics of *Blastocystis *sp. subtype 3 [44,48]. Patients who were singly infected with *Blastocystis *were as likely to show symptoms as those singly infected with *Cryptosporidium*. As such, the increase could have represented the emergence of a new subtype of *Blastocystis *in the US population during the 1990's.

The distribution shown in current studies usually identifies subtype 3 as the most common, with subtype 1 found at a frequency of 10–60% of subtype 3. This pattern has been found in varying geographic regions, such as China, Greece, Denmark, and Singapore. While this may be a statistical anomaly, the etiology of this relationship may be of interest.

### Transmission mechanisms

Regardless of opinions concerning pathogenicity, the existence of long-term trends in the prevalence of *Blastocystis *may be of scientific interest, especially when this occurred during a time when the frequency of detection of other enteric protozoa, such as *Giardia lamblia *and *Entamoeba histolytica *decreased [4,14]. Rising and falling prevalences have been found in propagated source epidemics such as influenza, but these trends occur over shorter time periods. A multi-year trend was reported in Scotland in association with epidemic infection of farm animals with *Salmonella typhimurium *and subsequent zoonotic transmission to the population [112]. The question of how *Blastocystis *is able to spread in industrialized countries with modern water treatment facilities remains to be addressed, but researchers have reported potential vectors as contaminated produce [113], sewage effluent [114], and contamination of treated drinking water due to infiltration at points distant from the treatment facility [115].

### Global trends

One study noted that case reports of *Blastocystis *infection began to increase world-wide in 1984 [3]. Was the US trend part of a larger global increase? Studies concerning trends in *Blastocystis *infection are lacking, and many countries have historically used detection methods that have been found to have a low sensitivity [116]. Trends in *Blastocystis *infection may be mirrored in the prevalence of chronic lower gastrointestinal illness of unknown etiology. One study noted a statistically significant increase in IBS prevalence in younger patient groups [117], but studies of the prevalence of IBS are lacking as well. Rates of inflammatory bowel disease (IBD) are better documented, possibly because they result in hospital admissions. Researchers noted increases of 25–100% in the incidence or rates of hospitalization for IBD during the 1990's in the United States [118], Ireland [119], Scotland [120] and Italy [121] but not in Canada [122] or Minnesota [123]. In some areas, the increase was most pronounced in individuals 18-years old and younger, with a doubling of the incidence of IBD in Scotland in individuals aged 12–18 years [120]. It is possible that other factors could have contributed to an increase in chronic gastrointestinal illness of unknown etiology during this time period. Researchers have cited the stress of city living [21] and the stress of international travel [124] as factors in development of such illness so it is possible that increased urbanization and travel has lead to greater morbidity. Serious gastrointestinal illness has been attributed to various viral and bacterial causes [125] and the hygiene hypothesis, which suggests that lack of gastrointestinal infection in childhood leads to chronic gastrointestinal illness [126]. Additional research may be valuable in understanding the relationship between trends in the prevalence of unexplained chronic gastrointestinal illness and poorly understood protozoal infections.

### Pathogenesis

Studies of the pathogenesis of *Blastocystis *have focused on immunological reactions of epithelial cells to proteases secreted by *Blastocystis*. Co-culture studies of *Blastocystis *have shown *Blastocystis *initially down-regulates and then up-regulates production of the inflammatory cytokine IL-8 in epithelial cells [127].

Specific *in vitro *co-culture studies have indicated that *Blastocystis *possesses pathogenicity mechanisms [83,128]. Pathogenesis was reported to result from interaction between parasite products (e.g. cysteine protease from the zoonotic isolate *Blastocystis ratti *WR1) and enterocytes that influence host inflammatory and immunological responses. *Blastocystis ratti *WR1 cysteine protease upregulated interleukin IL-8 gene expression through nuclear factor κB activation, but metronidazole treatment averted IL-8 production. *Blastocystis *also induced cell apoptosis, possibly in a subtype-dependent manner. An *in vitro *study also reported that the activation of nuclear factor κB/inhibitor of κB (NF-κB/IκB) signaling system lead to production of pro-inflammatory mediators, including IL-8, regulated upon activation, normal T-cell expressed, and secreted (RANTES) chemokines, and transforming-growth-factor TGF-β resulting in intestinal inflammation [83].

The pathogenesis of *Blastocystis *has been problematic, since the infection presents with abdominal pain in the absence of endoscopic findings [129]. Recent research into the etiology of abdominal pain, constipation and diarrhea in patients with symptoms attributed to IBS has suggested that the these symptoms are produced by high levels of serine protease produced in the gastrointestinal tract which are capable of exciting neurons directly through the protease-activated receptor-2 (PAR2) pathway [84,89]. This may offer an explanation for the conundrum of patients who experience pain in gastrointestinal illness in the absence of endoscopic findings [129].

While *Blastocystis *infection may be more serious in immunocompromised patients [130], studies have found that the majority of patients with symptomatic *Blastocystis *infection are immunocompetent, a pattern that is also present in infection with *Giardia *and *Entamoeba histolytica*. A Canadian study found that of 103 symptomatic patients with findings of only *Blastocystis *infection, only 3 were immunocompromised. Outbreaks of symptomatic *Blastocystis *infection have occurred in demographic groups not traditionally associated with immunocompromised status, such as groups of schoolchildren in the Middle East [58] and parents and children from the same families [131,132].

### Conflicting research results

To better understand conflicting findings concerning *Blastocystis *pathogenicity, we surveyed studies in the US National Institutes of Health Pubmed Database (see additional file 1). Studies reported pathogenicity in 84% (86/102) of the cases, and non-pathogenicity in 16% (16/102).

Many researchers identified a specific finding which they felt would be inconsistent with the behavior of a pathogen, such as a lack of correlation between the quantity of *Blastocystis *in stool samples and the patient's symptoms [133] or the absence of endoscopic findings in symptomatic infection [129].

Appendix B (additional file 2) summarizes characteristics of known gastrointestinal pathogens which may be of value. Additional observations follow, with details in additional file 3:

(1) All studies (16/16) finding *Blastocystis *to be non-pathogenic were conducted on subjects from more affluent countries;

(2) All studies finding *Blastocystis *to be non-pathogenic performed after 1994 used FECT or MIF for detection [134-136];

(2) Findings of non-pathogenicity were much more likely to have been reported by researchers studying *Blastocystis *in North America before 1994 (additional file 3, P < 0.0022, Fisher's Exact Test);

(3) North American studies reporting pathogenicity were often conducted in laboratories in coastal cities (Vancouver, New York, Palo Alto) which served individuals with travel history to less developed countries [6] or processed large numbers of samples (> 2,500) from the community [10,111]. Studies conducted on the more insular population of a health maintenance organization found *Blastocystis *to be non-pathogenic [5,9,137-140].

**Figure 8:**
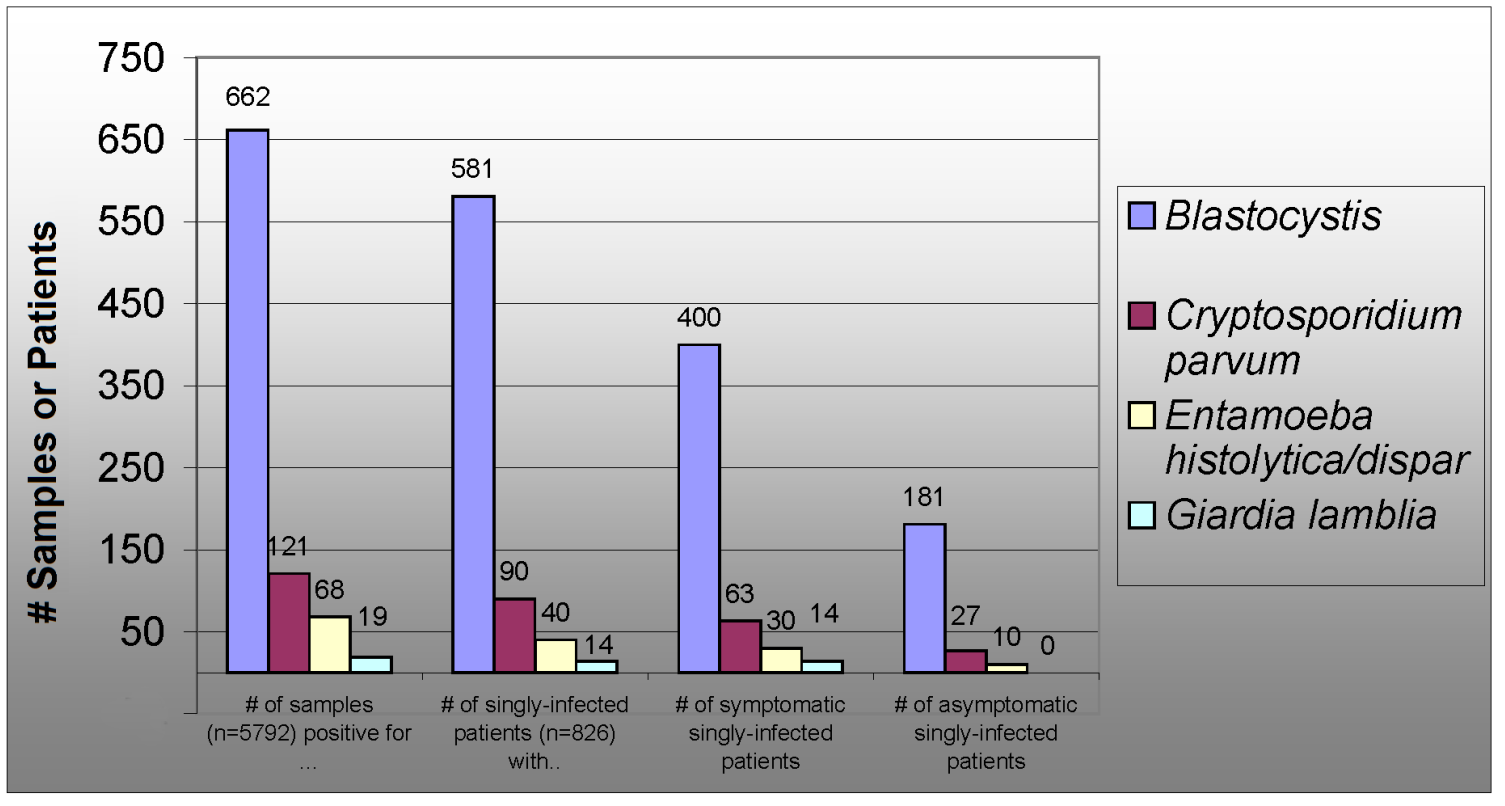
**The characteristics of common enteric protozoa reported in study of 5792 specimens from US patients collected in 2000**[**14**]. Studies from the 1980's reported *Blastocystis *was usually found as a co-infection with *Giardia *or *Entamoeba histolytica *in symptomatic patients [139,140], which was not the case in 2000 [14]. In 2000, the number of symptomatic patients who were found to be singly infected with *Blastocystis *(400) exceeded the number of samples found positive for *Cryptosporidium*, *Giardia*, and *Entamoeba histolytica *combined. Patients singly infected with *Blastocystis *were as likely to be symptomatic as patients singly infected with Cryptosporidium (69% vs. 70%) [14].

The variable sensitivity of diagnostic techniques discussed earlier may be responsible for some of the disagreement. Additional causes may include:

***Blastocystis *sp. subtype 2 may have been the dominant genotype in the United States in the early 1990's** : During this time, immigrants from Southeast Asia had a high rate of infection which was difficult to detect [109]. The description of *Blastocystis *as a co-infection in symptomatic patients from Californian physicians in the 1980's [139] is consistent with the behavior of *Blastocystis *sp. subtype 2 [44]. *Blastocystis *acquired overseas was associated with symptomatic infection, while US-acquired blastocystosis was asymptomatic [6]. Following an increase in detection rates of *Blastocystis *in the 1990's (see Appendix A in additional file 1), US studies now show *Blastocystis *appearing frequently as a symptomatic mono-infection [14] with characteristics similar to *Cryptosporidium parvum *and *Entamoeba histolytica*. (Figure 8).

**Symptomatic carriage of enteric protozoa may be mediated by host genetics (see Appendix B in additional file**2**)** : In affluent countries, if symptomatic carriers can seek and receive treatment, they may leave a population of asymptomatic carriers to be studied by epidemiologists.

Studies may have fulfilled many criteria listed in a 1990 communication as necessary to establish the pathogenicity of *Blastocystis *[110]: fulfillment of Koch's postulates [48,104,141]; identification of an immune response [82,85,86]; definition of the pathogenesis [83,127,128,142]; identification of treatments [94,143,144]; and description of point source outbreak of diarrhea where *Blastocystis *was the cause [57,131,145].

## Conclusion

1. *Blastocystis *comprises a group of genetically diverse organisms, some of which will cause chronic or acute infection in some immunocompetent humans. The behavior of *Blastocystis *in humans is consistent with that of *Giardia *and *Entamoeba histolytica *– expression of symptoms depends on parasite genotype, host genotype, host immunity, and age. Parasite genotype may vary geographically along with other factors.

2. Most diagnostics and treatments in clinical use for blastocystosis have been shown to have low sensitivity. Stool culture may provide the best short-term option for improving sensitivity, with serum antibody testing being a better option if it becomes available. PCR testing is currently the only way to identify the genotype of *Blastocystis *isolates.

3. Researchers from less affluent countries consistently report that *Blastocystis *is pathogenic, while some researchers in more affluent countries have authored contradictory studies. The outcome has been that little clinical research is performed or supported by affluent countries, while less affluent countries now publish sophisticated studies into *Blastocystis *infection [43,51,53,75,94,146,147].

4. *Blastocystis *has met all criteria for pathogenicity that are met by *Giardia *and *Entamoeba histolytica*, such as fulfillment Koch's Postulates [48,104,141,148], demonstration of a treatment that eliminates the organism and the symptoms, an immune response that is elevated in symptomatic individuals [36], and a positive association with symptoms in most studies. *Blastocystis *fails to meet criteria which *Giardia *and *Entamoeba histolytica *fail to meet. For example, all persons infected are not symptomatic; all researchers in all geographies have not shown an association between infection and symptoms; and the use of inadequate methods in some historical studies has produced the appearance of non-pathogenicity [149,150].

5. The lack of reliable diagnostics and the development of metronidazole resistance in *Blastocystis *may lead to many undiagnosed infections. *Blastocystis *may play a significant role in several chronic gastrointestinal illnesses of unknown etiology which can be expensive to manage and debilitating to patients.

## Abbreviations

ATCC: American Type Culture Collection; CWM: CONSED™ preservation followed by wet mounting; FECT: Formol Ethyl Acetate Concentration Technique; IBD: Inflammatory Bowel Disease; IBS: Irritable Bowel Syndrome; IBS-c: Constipation predominant Irritable Bowel Syndrome; IBS-d: Diarrhea predominant Irritable Bowel Syndrome; IFA: Indirect Immunofluorescence Assay; MIF: Merthiolate-Iodine Concentration; PAR2: Protease-Activated Receptor-2; PCR: Polymerase Chain Reaction; SAF: Sodium Acetate-acetic acid-formalin; SSU rRNA: Small subunit ribosomal RNA

## Competing interests

The authors declare that they have no competing interests.

## Authors' contributions

All authors engaged in developing the manuscript and approved the final version.

## Supplementary Material

Additional file 1 Appendix A – Additional charts. Click here for file

Additional file 2 Appendix B – Characteristics of known gastrointestinal pathogens. Click here for file

Additional file 3 List of *Blastocystis *studies categorized by researcher finding and geographic location. Click here for file
