# Supplementary material for: Oh my aching gut: irritable bowel syndrome, Blastocystis, and asymptomatic infection
Source: Parasit Vectors. 2008 Oct 21;1:40. doi: 10.1186/1756-3305-1-40 (PMC2627840; doi:10.1186/1756-3305-1-40)
Supplement: Additional file 1 — Appendix A – Additional charts. [file 1756-3305-1-40-S1.pdf]

APPENDIX A
Supplementary Figures and Graphs for Oh my aching gut: IBS, *Blastocystis* and asymptomatic infection
BMC Parasites and Vectors, 2008

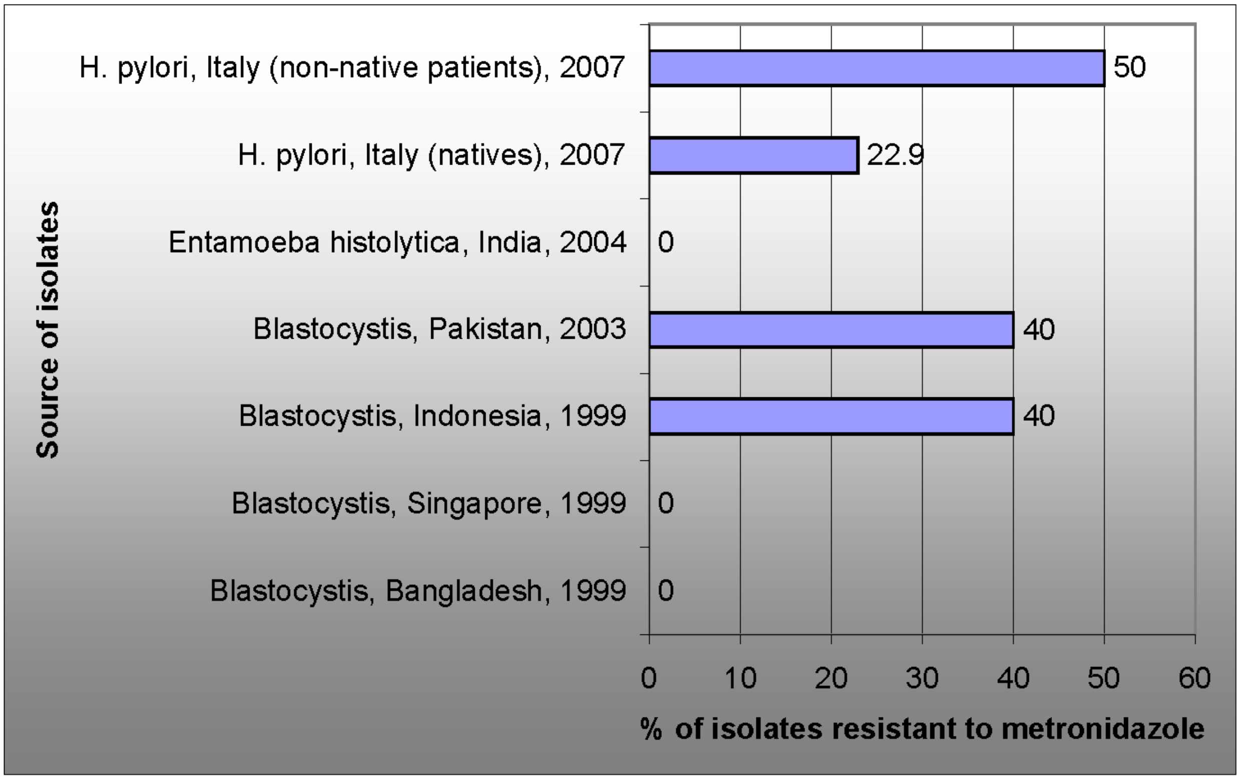

Figure A-1: Metronidazole resistance in common enteric organisms: *Blastocystis* [1] [2], *Entamoeba histolytica* [3], and *Helicobacter pylori* [4]. *Blastocystis* isolates from Pakistan were found to be resistant to furazolidone and ciproflaxin [5].

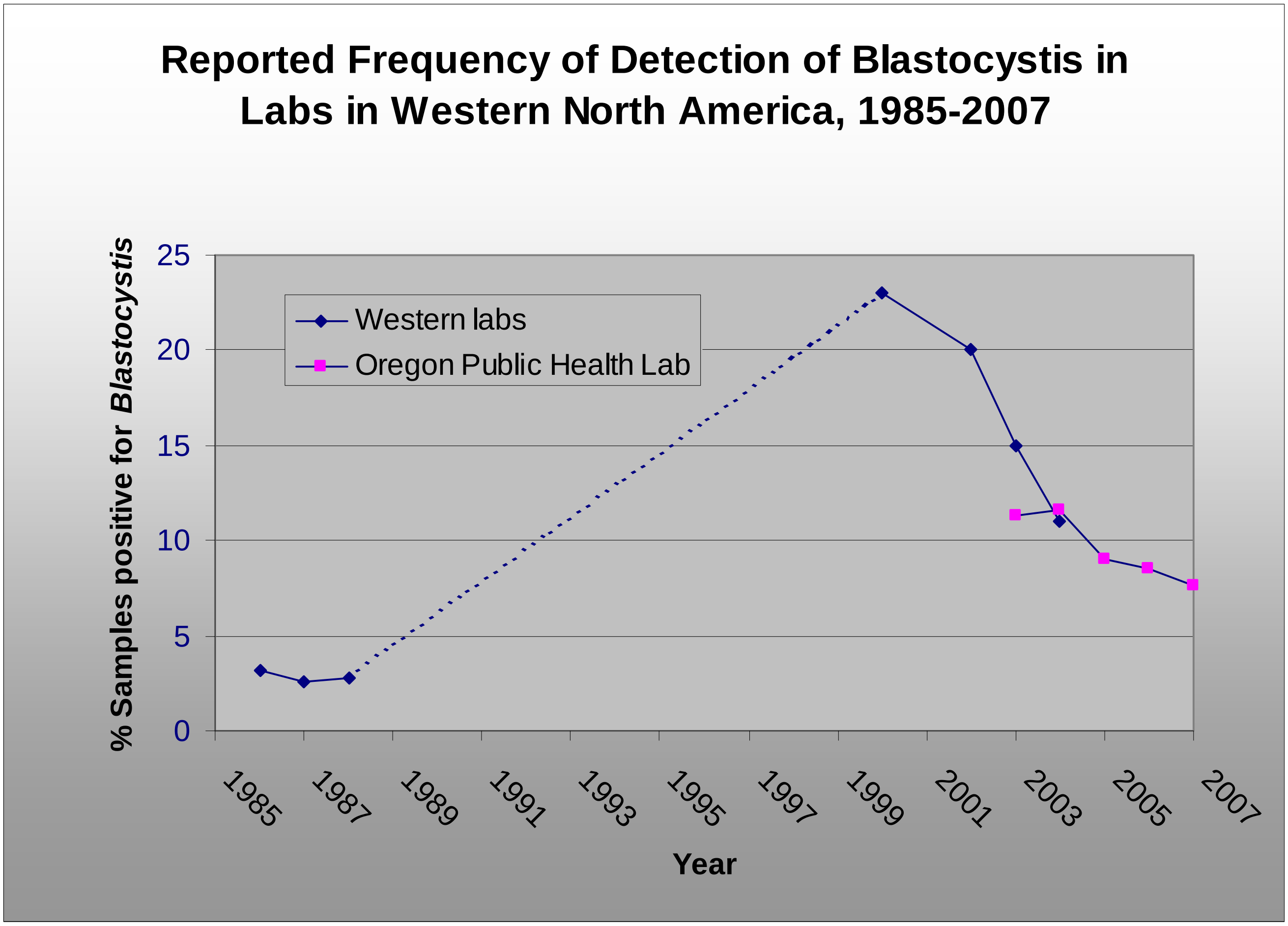

Figure A-2 – Frequency of detection of *Blastocystis* reported from laboratories from the Western United States and Canada. 1987 data is from US-wide study [6], 1988 from California [7], 1990 from Vancouver BC [8], 2000-2004 data is from US-wide studies that emphasized samples from Western US [9, 10]. Data in purple was obtained from the Oregon Public Health Laboratory for reference.

Data for Figure A-2

Published Data

Year Studied	Location	% Samples Positive	Author
1986-1987	Vancouver, BC	3.2	Doyle, 1990
1987	US	2.6	Kappus, 1991
1987-1988	Palo Alto, CA	2.8	Babb, 1989
2000	Arizona (US-wide)	23	Amin, 2002
2002	Arizona (US-wide)	20	Amin, 2006
2003	Arizona (US-wide)	15	Amin, 2006
2004	Arizona (US-wide)	11	Amin, 2006

Studies from Amin were performed at a clinical laboratory in Arizona in the Western US. The studies incorporated samples from many US states, but samples from the Western US dominate the studies.

Unpublished Data – Personal Communication Oregon State Public Health Laboratory

Year	% Samples Positive	N samp	N pos
2003 (partial)	11.3	884	100
2004	11.6	925	107
2005	9.0	980	88
2006	8.5	797	68
2007 (partial)	7.6	460	35

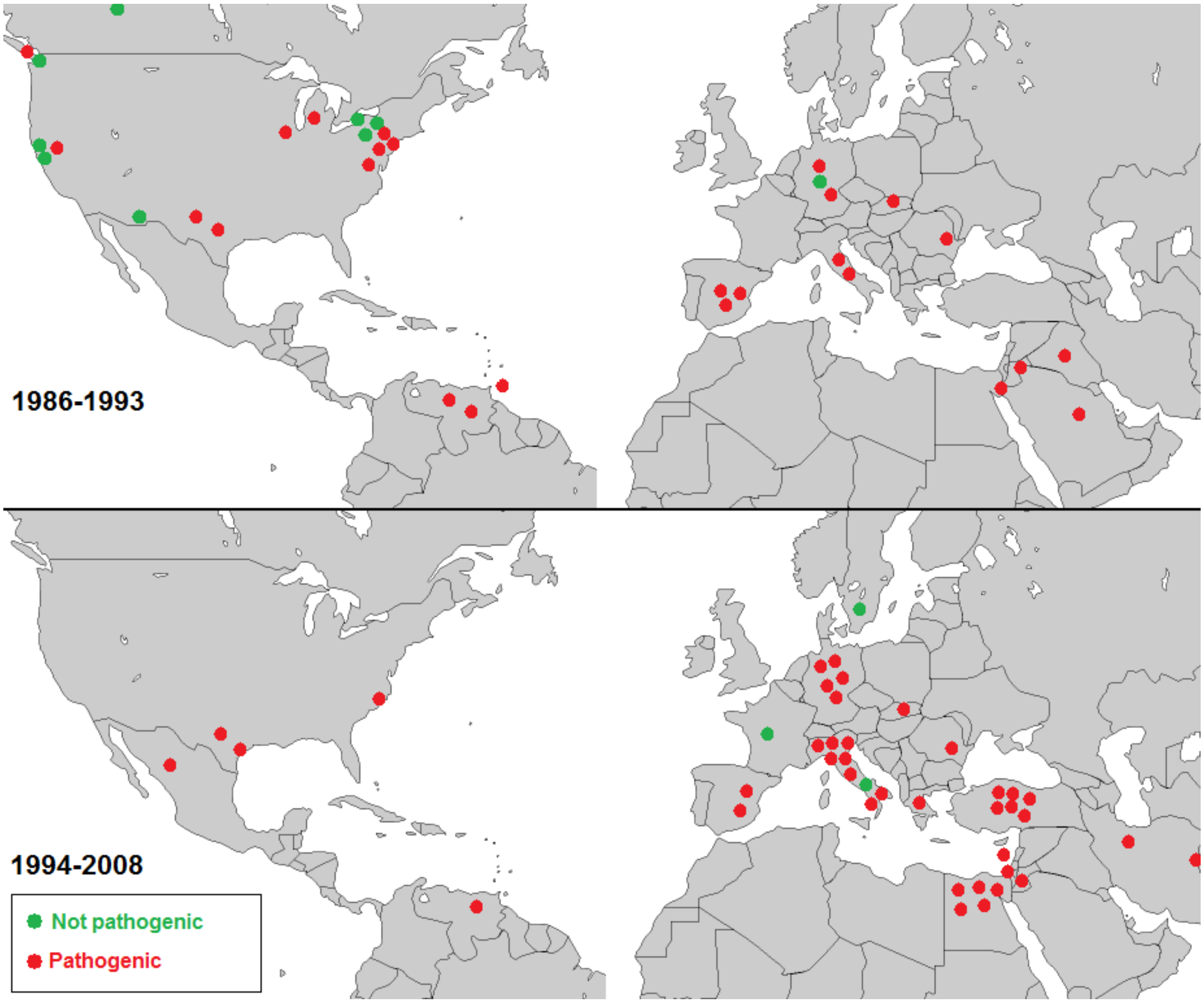

Figure A-3: Comparison of researcher findings concerning association of *Blastocystis* with symptoms during two time periods, 1986-1994 and 1994-2008. In the later period, only three studies in this region found *Blastocystis* to be unassociated with disease. All three of these studies used the FECT detection method, which has been found to be more sensitive to a type of *Blastocystis* which has been associated with asymptomatic infection.

State	Frequency of detection of *Blastocystis* (n samples/n positive)
New York	24.7% (62/251)
New Mexico	22% (36/164)
Maine	20.3% (57/280)
Oregon	17.1% (48/281)
California	20% (263/1328)
Texas	7.2% (13/181)
Illinois	5.3% (21/396)

Table A-1: Frequency of detection of *Blastocystis* by CWM from one US clinical laboratory in samples from various US states, in 2002-2004 [10]. The study suggested that prevalence was higher in coastal states and lower in landlocked states. While climatic conditions may play a role, it is possible other regional factors may be responsible.

Country	Researcher, Year	Type of assay	Findings
USA	Zierdt, 1993 [11]	Serum ELISA antibody	All patients diagnosed with blastocystosis were positive at titers of 1:50 and higher; all members of the control group (blood donors) were negative at titer of 1:50
USA	Zierdt, 1993 [11]	Serum indirect-immunofluorescence assay (IFA)	Patients diagnosed with blastocystosis were all positive at titers above 1:100; control group (blood donors) was positive only at titers of 1:10, 1:20, and 1:50
Pakistan	Hussein, 1997 [12]	Serum ELISA antibody	Significantly elevated response in IBS patients
Japan	Kaneda, 2003 [13]	Serum IFA antibody	70% of asymptomatic carriers serologically positive by IFA; serum dilutions greater than 1:60 failed to produce response in all asymptomatic carriers.
Egypt	Mahmoud, 2003 [14]	Serum ELISA antibody	83.3% of blastocystosis patients were positive at a titer of 1:800; 0% of asymptomatic carriers positive at this titer
Egypt	Mahmoud, 2003 [14]	Fecal IgA antibody	100% of blastocystosis patients were positive at a titer of 1:400; 0% of asymptomatic carriers positive at this titer

Table A-2: Summary of results from serum antibody testing in *Blastocystis* infection

References:

1. Haresh K, Suresh K, Khairul Anus A, Saminathan S: **Isolate resistance of Blastocystis hominis to metronidazole**. *Trop Med Int Health* 1999, **4**(4):274-277.
2. Yakoob J, Jafri W, Jafri N, Islam M, Asim Beg M: **In vitro susceptibility of Blastocystis hominis isolated from patients with irritable bowel syndrome**. *Br J Biomed Sci* 2004, **61**(2):75-77.
3. Bansal D, Sehgal R, Chawla Y, Mahajan RC, Malla N: **In vitro activity of antiamoebic drugs against clinical isolates of Entamoeba histolytica and Entamoeba dispar**. *Ann Clin Microbiol Antimicrob* 2004, **3**:27.
4. Zullo A, Perna F, Hassan C, Ricci C, Saracino I, Morini S, Vaira D: **Primary antibiotic resistance in Helicobacter pylori strains isolated in northern and central Italy**. *Aliment Pharmacol Ther* 2007, **25**(12):1429-1434.
5. Yakoob J, Jafri W, Jafri N, Khan R, Islam M, Beg MA, Zaman V: **Irritable bowel syndrome: in search of an etiology: role of Blastocystis hominis**. *Am J Trop Med Hyg* 2004, **70**(4):383-385.
6. Kappus KK, Juranek DD, Roberts JM: **Results of testing for intestinal parasites by state diagnostic laboratories, United States, 1987**. *MMWR CDC Surveill Summ* 1991, **40**(4):25-45.
7. Babb RR, Wagener S: **Blastocystis hominis--a potential intestinal pathogen**. *West J Med* 1989, **151**(5):518-519.
8. Doyle PW, Helgason MM, Mathias RG, Proctor EM: **Epidemiology and pathogenicity of Blastocystis hominis**. *J Clin Microbiol* 1990, **28**(1):116-121.
9. Amin OM: **Seasonal prevalence of intestinal parasites in the United States during 2000**. *Am J Trop Med Hyg* 2002, **66**(6):799-803.
10. Amin O: **Epidemiology of Blastocystis hominis in the United States**. *Research Journal of Parasitology* 2005, **2006**(1 (1)):1-10.
11. Zierdt CH, Nagy B: **Antibody response to Blastocystis hominis infections**. *Ann Intern Med* 1993, **118**(12):985-986.
12. Hussein EM, Hussein AM, Eida MM, Atwa MM: **Pathophysiological variability of different genotypes of human Blastocystis hominis Egyptian isolates in experimentally infected rats**. *Parasitol Res* 2008, **102**(5):853-860.
13. Kaneda Y, Horiki N, Cheng X, Tachibana H, Tsutsumi Y: **Serologic response to Blastocystis hominis infection in asymptomatic individuals**. *Tokai J Exp Clin Med* 2000, **25**(2):51-56.
14. Mahmoud MS, Saleh WA: **Secretory and humoral antibody responses to Blastocystis hominis in symptomatic and asymptomatic human infections**. *J Egypt Soc Parasitol* 2003, **33**(1):13-30.
