# Supplementary material for: Oh my aching gut: irritable bowel syndrome, Blastocystis, and asymptomatic infection
Source: Parasit Vectors. 2008 Oct 21;1:40. doi: 10.1186/1756-3305-1-40 (PMC2627840; doi:10.1186/1756-3305-1-40)
Supplement: Additional file 2 — Appendix B – Characteristics of known gastrointestinal pathogens. [file 1756-3305-1-40-S2.pdf]

APPENDIX B

Characteristics of Known Enteric Pathogens and Diseases

Oh my aching gut: IBS, *Blastocystis*, and asymptomatic infection
BMC Parasites and Vectors, 2008

This collection of characteristics of established enteric pathogens may be of value in understanding what behavior would be expected from new enteric pathogens.

Prevalence of enteric pathogens in asymptomatic indivividuals

1. In Egypt, 21% of asymptomatic individuals have been found to carry *Entamoeba histolytica*, while 24% carry *Entamoeba dispar* [1].
2. In Greece, among asymptomatic individuals, less than 1% were found to carry *E. histolytica* while 8% carried *E. dispar* [1].
3. In a study of a slum in Brazil, 11% carried *E. histolytica,* and 9% carried *E. dispar* [1].
4. Immunocompetent individuals infected with the identical genotype of *E. histolytica* can exhibit very different clinical presentations [1].

Host genotype and expression of symptoms in enteric infections

1. Researchers have suggested that symptoms seen in disease result from the combination of infection with host genetic factors.
2. Infection with *Helicobacter pylori* is asymptomatic in many individuals, but polymorphisms that influence production of the cytokine IL-1 can predispose infected individuals toward the development of gastric cancer [2].
3. Asymptomatic carriers of *Vibrio cholerae* exist. The same polymorphisms which confer immunity to symptoms in *Vibro cholerae* infection have been found to be responsible for the development of cystic fibrosis [3].
4. Certain blood types may be correlated with symptomatic and asymptomatic gastrointestinal infection [4].
5. Additional polymorphisms may be responsible for producing the wide range of symptoms seen in infection with *E. histolytica* [1].
6. The genetic polymorphisms that confer susceptibility to disease in items 2-5 above are common and carried by healthy individuals.
7. In fact, in Zulu, the word for the disease caused by amoebic liver abscess is *isigwebedhla*, which means a disease of strong young men [1].
8. Mice are generally immune to *E. histolytica*, but that immunity is lost in IL-10 knockout mice [5]. IL-10 is a cytokine with immunosuppressive (anti-inflammatory) activity.
9. Some studies have suggested that HIV status is not a significant factor in expression of symptoms with *E. histolytica* [6, 7].

10. A study of *E. histolytica* infection found that certain individuals who were likely to be exposed never developed symptoms and also never developed a serological response. The phenomenon was correlated with family lineage, suggesting a genetic link [8, 9].
11. Variation can exist between ethnic groups in the prevalence of polymorphisms [10]. Some Mexican populations carry a gene which increases the risk of the development of amoebic liver abscess [1].

TNF-Alpha in *Entamoeba histolytica* infection

1. TNF-α is an important component of the immune system, and provides protection against bacterial infection and some types of cancer. The use of anti-inflammatory medications that inhibit production of TNF-α can reactivate viral and bacterial infections in patients [11].
2. But the use of a TNF-α inhibitor reduces the severity of infection with *E. histolytica* in mice [12].
3. TNF-α is a chemo-attractant for *E. histolytica* [13].

Serotonin in enteric infections

1. Researchers have suggested serotonin may play a role in host immune response in enteric infections [14].
2. *E. histolytica* has been found to induce serotonin secretion in enteric epithelial cells [15].
3. Serotonin has been found to increase the activity of *E. histolytica* infection *in vitro* [16] and the severity of infection *in vivo* [17].
4. Patients with symptomatic infection with *E. histolytica* can show elevated serum levels of serotonin [18].

Irritable bowel syndrome

1. IBS patients exhibit polymorphisms that cause high TNF-α production, slow serotonin uptake, and low IL-10 production [19, 20].
2. The prevalence of IBS in Mexico City is 35% [21].
3. US citizens returning from international travel exhibit a rate of new-onset persistent gastrointestinal illness or IBS of more than 39% (27/68) [22].
4. Selective serotonin re-uptake inhibitors, which are sometimes used in the treatment of IBS, inhibit production of TNF-α and increase production of IL-10 [23, 24].

Age in expression of symptoms in enteric protozoal infections

1. Symptomatic infection with *E. histolytica* is more common in adults than children [25, 26] .
2. *Giardia lamblia* assemblage B has been found to be correlated with asymptomatic infection in children, but not adults [27].

3. IBS and blastocystosis are more common in adults, especially those aged 30-50 years [28-31].
4. Children produce lower levels of inflammatory cytokines [32]. These cytokines stimulate production of TNF-α.

Counter-intuitive findings in epidemiological studies

1. In a study of a slum in Brazil, the pathogenic *E. histolytica* was actually found more frequently than the non-pathogenic *E. dispar* in asymptomatic individuals [1].
2. Physicians have reported the appearance of groups of individuals in whom infection with *Giardia* and *E. histolytica* is not correlated with symptoms [33, 34].
3. A study of homosexuals in San Francisco found infection with *Giardia* and *E. histolytica* was uncorrelated with symptoms [35].
4. Studies by the same researcher found that *Blastocystis* infection was uncorrelated with symptoms [36-38].
5. In general, there is a poor correlation between the ability to detect protozoal infections and the patient's symptomatic status. This is true in *Giardia lamblia* infection [39], *E. histolytica* infection, and *Cryptosporidium* infection [40].

Treatment of enteric pathogens

1. The ability of organisms to develop metronidazole resistance varies substantially between different species [41].
2. Some enteric protozoal infections have few treatment options – *Cyclospora* and C*ryptosporidium* do not respond to many antiprotozoal drugs that are used successfully in the treatment of other protozoal infections [42].
3. *In vitro* study has found iodoquinol to be one of the least effective drugs against *Blastocystis* infection [29].
4. In enteric infections, treatment with an anti-infective can improve symptoms without eradicating the pathogen responsible for the symptoms [43].

Symptoms of infection with enteric pathogens

1. *E. histolytica* can present with abdominal pain and constipation, rather than diarrhea [26].
2. Abdominal pain and constipation are the most common symptoms of blastocystosis, not diarrhea or fever [44].
3. Prior study has concluded that *Blastocystis* is non-pathogenic because infection is not associated with visible damage in endoscopy [45].
4. Additionally, IBS is considered a functional disorder because of lack of findings in endoscopy, X-rays and blood tests [46].
5. Colonic tissue samples from IBS patients have been found to produce a high level of a serine protease which produces symptoms associated with IBS when introduced into mice [47]. The study was unable to identify the source of the protease.

6. Proteases may influence gastrointestinal motility through protease-activated receptor-2 activation [48].

Enteric disease and psychiatric symptoms

1. Infection with *E. histolytica* has been associated with a psychiatric condition called "amoebic neurosis" [49].
2. Soldiers returning from World War II exhibited psychiatric symptoms and "debility" which was associated with long-term *E. histolytica* infection [50]. Symptoms subsided following antiprotozoal treatment. The disease was most prevalent in army units deployed in the Middle East.
3. The symptoms of widespread pain, diarrhea and weight loss in IBS have been presumed to be an expression of somatic illness. Psychiatrists have suggested the symptoms are related to sexual abuse [51] or the accumulation of life's stresses [52].
4. Subsequent research has found that the colonic tissue in IBS patients produces high levels of a serine protease which activates nerve cells directly through the protease-activated receptor-2 (PAR2) pathway [47].
5. The proteases were produced by biopsies from patients with constipation-predominant IBS, diarrhea-predominant IBS, and inflammatory bowel disease (IBD).
6. PAR2 was the second member of a series of PARs which was discovered in 1994 through analysis of the human genomic library [53].
7. PAR2 receptors are present on a variety of cells in the human body, including cells in the gastrointestinal tract, pancreas, kidney, liver, airway, prostate, ovary, and eye, smooth muscle, T cell lines, neutrophils, and certain tumor cell lines [53].
8. Elevated serine protease levels are found in patients with diarrhea predominant IBS, but not patients with infectious viral or bacterial diarrhea [54].
9. PAR2 activation has been implicated in the pathogenesis of *Helicobacter pylori* infection [55] and PAR2 gene expression is enhanced in infection with *E. histolytica* [56].

References:

1. Stanley SL, Jr.: **Amoebiasis**. *Lancet* 2003, **361**(9362):1025-1034.
2. El-Omar EM, Carrington M, Chow WH, McColl KE, Bream JH, Young HA, Herrera J, Lissowska J, Yuan CC, Rothman N *et al*: **The role of interleukin-1 polymorphisms in the pathogenesis of gastric cancer**. *Nature* 2001, **412**(6842):99.
3. Gabriel SE, Brigman KN, Koller BH, Boucher RC, Stutts MJ: **Cystic fibrosis heterozygote resistance to cholera toxin in the cystic fibrosis mouse model**. *Science* 1994, **266**(5182):107-109.
4. Haque R, Mondal D, Kirkpatrick BD, Akther S, Farr BM, Sack RB, Petri WA, Jr.: **Epidemiologic and clinical characteristics of acute diarrhea with emphasis on Entamoeba histolytica infections in preschool children in an urban slum of Dhaka, Bangladesh**. *Am J Trop Med Hyg* 2003, **69**(4):398-405.
5. Hamano S, Asgharpour A, Stroup SE, Wynn TA, Leiter EH, Houpt E: **Resistance of C57BL/6 mice to amoebiasis is mediated by nonhemopoietic cells but requires hemopoietic IL-10 production**. *J Immunol* 2006, **177**(2):1208-1213.
6. Moran P, Ramos F, Ramiro M, Curiel O, Gonzalez E, Valadez A, Gomez A, Garcia G, Melendro EI, Ximenez C: **Infection by human immunodeficiency virus-1 is not a risk factor for amebiasis**. *Am J Trop Med Hyg* 2005, **73**(2):296-300.
7. Church DL, Sutherland LR, Gill MJ, Visser ND, Kelly JK: **Absence of an association between enteric parasites in the manifestations and pathogenesis of HIV enteropathy in gay men. The GI/HIV Study Group**. *Scand J Infect Dis* 1992, **24**(5):567-575.
8. Duggal P, Haque R, Roy S, Mondal D, Sack RB, Farr BM, Beaty TH, Petri WA, Jr.: **Influence of human leukocyte antigen class II alleles on susceptibility to Entamoeba histolytica infection in Bangladeshi children**. *J Infect Dis* 2004, **189**(3):520-526.
9. Haque R, Mondal D, Duggal P, Kabir M, Roy S, Farr BM, Sack RB, Petri WA, Jr.: **Entamoeba histolytica infection in children and protection from subsequent amebiasis**. *Infect Immun* 2006, **74**(2):904-909.
10. Netsawang J, Tangwattanachuleeporn M, Hirankarn N, Wongpiyabovorn J: **The distribution of IL-10 promoter polymorphism in Thais**. *J Med Assoc Thai* 2004, **87 Suppl 2**:S117-122.
11. Brassard P, Kezouh A, Suissa S: **Antirheumatic drugs and the risk of tuberculosis**. *Clin Infect Dis* 2006, **43**(6):717-722.
12. Zhang Z, Mahajan S, Zhang X, Stanley SL, Jr.: **Tumor necrosis factor alpha is a key mediator of gut inflammation seen in amebic colitis in human intestine in the SCID mouse-human intestinal xenograft model of disease**. *Infect Immun* 2003, **71**(9):5355-5359.
13. Blazquez S, Zimmer C, Guigon G, Olivo-Marin JC, Guillen N, Labruyere E: **Human tumor necrosis factor is a chemoattractant for the parasite Entamoeba histolytica**. *Infect Immun* 2006, **74**(2):1407-1411.
14. Yang GB, Lackner AA: **Proximity between 5-HT secreting enteroendocrine cells and lymphocytes in the gut mucosa of rhesus macaques (Macaca mulatta) is suggestive of a role for enterochromaffin cell 5-HT in mucosal immunity**. *J Neuroimmunol* 2004, **146**(1-2):46-49.

15. McGowan K, Kane A, Asarkof N, Wicks J, Guerina V, Kellum J, Baron S, Gintzler AR, Donowitz M: **Entamoeba histolytica causes intestinal secretion: role of serotonin**. *Science* 1983, **221**(4612):762-764.
16. Ahmed I, Khan MA, Sen PC: **In vitro effect of 5-hydroxytryptamine on concanavalin-A agglutinability and multiplication of Entamoeba histolytica**. *Indian J Exp Biol* 1993, **31**(3):285-287.
17. Acharya DP, Sen MR, Sen PC: **Effect of exogenous 5-hydroxytryptamine on pathogenicity of Entamoeba histolytica in experimental animals**. *Indian J Exp Biol* 1989, **27**(8):718-720.
18. Banu N, al. e: **Neurohumoral alterations and their role in amoebiasis**. *Indian J Clin Biochem* 2005, **20**(2):142-145.
19. Park MI, Camilleri M: **Genetics and genotypes in irritable bowel syndrome: implications for diagnosis and treatment**. *Gastroenterol Clin North Am* 2005, **34**(2):305-317.
20. van der Veek PP, van den Berg M, de Kroon YE, Verspaget HW, Masclee AA: **Role of tumor necrosis factor-alpha and interleukin-10 gene polymorphisms in irritable bowel syndrome**. *Am J Gastroenterol* 2005, **100**(11):2510-2516.
21. Schmulson M, Ortiz O, Santiago-Lomeli M, Gutierrez-Reyes G, Gutierrez-Ruiz MC, Robles-Diaz G, Morgan D: **Frequency of functional bowel disorders among healthy volunteers in Mexico City**. *Dig Dis* 2006, **24**(3-4):342-347.
22. Tuteja AK, Talley NJ, Gelman SS, Alder SC, Thompson C, Tolman K, Hale DC: **Development of functional diarrhea, constipation, irritable bowel syndrome, and dyspepsia during and after traveling outside the USA**. *Dig Dis Sci* 2008, **53**(1):271-276.
23. Maes M: **The immunoregulatory effects of antidepressants**. *Hum Psychopharmacol* 2001, **16**(1):95-103.
24. Diamond M, Kelly JP, Connor TJ: **Antidepressants suppress production of the Th1 cytokine interferon-gamma, independent of monoamine transporter blockade**. *Eur Neuropsychopharmacol* 2006, **16**(7):481-490.
25. Oyerinde JP, Ogunbi O, Alonge AA: **Age and sex distribution of infections with Entamoeba histolytica and Giardia intestinalis in the Lagos population**. *Int J Epidemiol* 1977, **6**(3):231-234.
26. Haque R, Huston CD, Hughes M, Houpt E, Petri WA, Jr.: **Amebiasis**. *N Engl J Med* 2003, **348**(16):1565-1573.
27. Sahagun J, Clavel A, Goni P, Seral C, Llorente MT, Castillo FJ, Capilla S, Arias A, Gomez-Lus R: **Correlation between the presence of symptoms and the Giardia duodenalis genotype**. *Eur J Clin Microbiol Infect Dis* 2008, **27**(1):81-83.
28. Wilson S, Roberts L, Roalfe A, Bridge P, Singh S: **Prevalence of irritable bowel syndrome: a community survey**. *Br J Gen Pract* 2004, **54**(504):495-502.
29. Doyle PW, Helgason MM, Mathias RG, Proctor EM: **Epidemiology and pathogenicity of Blastocystis hominis**. *J Clin Microbiol* 1990, **28**(1):116-121.
30. Amin O: **Epidemiology of Blastocystis hominis in the United States**. *Research Journal of Parasitology* 2005, **2006**(1 (1)):1-10.
31. Carbajal JA, Villar J, Lanuza MD, Esteban JG, Munoz C, Borras R: **[Clinical significance of Blastocystis hominis infection: epidemiologic study]**. *Med Clin (Barc)* 1997, **108**(16):608-612.

32. Duramad P, McMahon CW, Hubbard A, Eskenazi B, Holland NT: **Flow cytometric detection of intracellular TH1/TH2 cytokines using whole blood: validation of immunologic biomarker for use in epidemiologic studies**. *Cancer Epidemiol Biomarkers Prev* 2004, **13**(9):1452-1458.
33. Allason-Jones E, Mindel A, Sargeaunt P, Williams P: **Entamoeba histolytica as a commensal intestinal parasite in homosexual men**. *N Engl J Med* 1986, **315**(6):353-356.
34. Anand BS, Tuteja AK, Kaur M, Alam SM, Aggarwal DS, Mehta SP, Baveja UK: **Entamoeba histolytica cyst passers. Clinical profile and spontaneous eradication of infection**. *Dig Dis Sci* 1993, **38**(10):1825-1830.
35. Markell EK, Havens RF, Kuritsubo RA, Wingerd J: **Intestinal protozoa in homosexual men of the San Francisco Bay area: prevalence and correlates of infection**. *Am J Trop Med Hyg* 1984, **33**(2):239-245.
36. Markell EK, Udkow MP: **Blastocystis hominis**. *West J Med* 1990, **152**(6):721.
37. Markell EK, Udkow MP: **Association of Blastocystis hominis with human disease**. *J Clin Microbiol* 1988, **26**(3):609-610.
38. Udkow MP, Markell EK: **Blastocystis hominis: prevalence in asymptomatic versus symptomatic hosts**. *J Infect Dis* 1993, **168**(1):242-244.
39. Hanson KL, Cartwright CP: **Use of an enzyme immunoassay does not eliminate the need to analyze multiple stool specimens for sensitive detection of Giardia lamblia**. *J Clin Microbiol* 2001, **39**(2):474-477.
40. Garcia LS, Shimizu RY, Bernard CN: **Detection of Giardia lamblia, Entamoeba histolytica/Entamoeba dispar, and Cryptosporidium parvum antigens in human fecal specimens using the triage parasite panel enzyme immunoassay**. *J Clin Microbiol* 2000, **38**(9):3337-3340.
41. Upcroft JA, Upcroft P: **Drug susceptibility testing of anaerobic protozoa**. *Antimicrob Agents Chemother* 2001, **45**(6):1810-1814.
42. Farthing MJ: **Treatment options for the eradication of intestinal protozoa**. *Nat Clin Pract Gastroenterol Hepatol* 2006, **3**(8):436-445.
43. Treiber G, Lambert JR: **The impact of Helicobacter pylori eradication on peptic ulcer healing**. *Am J Gastroenterol* 1998, **93**(7):1080-1084.
44. Qadri SM, al-Okaili GA, al-Dayel F: **Clinical significance of Blastocystis hominis**. *J Clin Microbiol* 1989, **27**(11):2407-2409.
45. Zuckerman MJ, Watts MT, Ho H, Meriano FV: **Blastocystis hominis infection and intestinal injury**. *Am J Med Sci* 1994, **308**(2):96-101.
46. **The Merck Manual of Medical Information** [http://www.merck.com/mmhe/sec09/ch129/ch129a.html]
47. Cenac N, Andrews CN, Holzhausen M, Chapman K, Cottrell G, Andrade-Gordon P, Steinhoff M, Barbara G, Beck P, Bunnett NW *et al*: **Role for protease activity in visceral pain in irritable bowel syndrome**. *J Clin Invest* 2007, **117**(3):636-647.
48. Ossovskaya VS, Bunnett NW: **Protease-activated receptors: contribution to physiology and disease**. *Physiol Rev* 2004, **84**(2):579-621.
49. Barrett-Connor E: **Amebiasis, today, in the United States**. *Calif Med* 1971, **114**(3):1-6.
50. Stout M, Duncan T: **War, Surgery, and Medicine**. In: *The Official History of New Zealand in the Second World War.* Historical Publications Branch, 1954, Wellington, pp. 485-491, http://www.nzetc.org/tm/scholarly/tei-WH2Surg-pt2-c1-2.html; 1954.

51. Creed F, Guthrie E, Ratcliffe J, Fernandes L, Rigby C, Tomenson B, Read N, Thompson DG: **Reported sexual abuse predicts impaired functioning but a good response to psychological treatments in patients with severe irritable bowel syndrome**. *Psychosom Med* 2005, **67**(3):490-499.
52. Crofford LJ: **Violence, stress, and somatic syndromes**. *Trauma Violence Abuse* 2007, **8**(3):299-313.
53. Dery O, Corvera CU, Steinhoff M, Bunnett NW: **Proteinase-activated receptors: novel mechanisms of signaling by serine proteases**. *Am J Physiol* 1998, **274**(6 Pt 1):C1429-1452.
54. Gecse K, Roka R, Ferrier L, Leveque M, Eutamene H, Cartier C, Ait-Belgnaoui A, Rosztoczy A, Izbeki F, Fioramonti J *et al*: **Increased faecal serine protease activity in diarrhoeic IBS patients: a colonic lumenal factor impairing colonic permeability and sensitivity**. *Gut* 2008, **57**(5):591-599.
55. Kajikawa H, Yoshida N, Katada K, Hirayama F, Handa O, Kokura S, Naito Y, Yoshikawa T: **Helicobacter pylori activates gastric epithelial cells to produce interleukin-8 via protease-activated receptor 2**. *Digestion* 2007, **76**(3-4):248-255.
56. Zhang Z, Stanley SL, Jr.: **Stereotypic and specific elements of the human colonic response to Entamoeba histolytica and Shigella flexneri**. *Cell Microbiol* 2004, **6**(6):535-554.
